# Editorial: Bioactive Natural Products from Microbes: Isolation, Characterization, Biosynthesis and Structure Modification

**DOI:** 10.3389/fchem.2022.883652

**Published:** 2022-03-22

**Authors:** Xiachang Wang, Yuanyuan Lu, Khaled A. Shaaban, Guojun Wang, Xuekui Xia, Youjuan Zhu

**Affiliations:** ^1^ Jiangsu Key Laboratory for Functional Substances of Chinese Medicine, Nanjing University of Chinese Medicine, Nanjing, China; ^2^ State Key Laboratory of Natural Medicines, China Pharmaceutical University, Nanjing, China; ^3^ Center for Pharmaceutical Research and Innovation, College of Pharmacy, University of Kentucky, Lexington, KY, United States; ^4^ Harbor Branch Oceanographic Institute, Florida Atlantic University, Fort Pierce, FL, United States; ^5^ Key Biosensor Laboratory of Shandong Province, Biology Institute, Qilu University of Technology (Shandong Academy of Sciences), Jinan, China

**Keywords:** microbes, natural product, structure elucidation and modification, activity evaluation, biosynthesis

Natural products have played an invaluable role in drug development, as about 24 percent of approved drugs belong to natural products or their derivative (Newman and Cragg, 2020). Compared with the limitation of medicinal resources such as plants and animals, microbes from soil, air, ocean and even endophytes, have now become a potential source for drug lead discovery, due to their abundant sources and characteristic biosynthetic pathways for novel bioactive compounds. The ability of all living organisms to biosynthesize endogenous, specialized small molecules is genetically encoded. The magnitude of biosynthetic gene clusters in microbial genome suggests that the secondary metabolite wealth of microbe is largely untapped. Mining algorithms and scalable expression platforms have greatly expanded access to the chemical repertoire of microbial secondary metabolites. This current Research Topic aims to discover bioactive natural products from microbes as drug leads, using new technologies such as metagenomics and gene mining to look for the breakthrough of new drug research and development. Overall, fourteen contributions were collected including one review and thirteen original articles, highlighting the importance of microbial natural products discovery for drug leads.

A review article by Yu et al. summarized the biological and chemical aspects of *Aspergillus niger* strains including their sources, BGCs, and secondary metabolites as well as biological properties and biosynthetic pathways. *A. niger* has become promising application products which possess a large number of cryptic biosynthetic gene clusters (BGCs) and produce various biomolecules as secondary metabolites with a broad spectrum of application fields including agriculture, food industry, and medicine. In the past 5 years, the number of new bioactive compounds from *A. niger* has been decreasing so that more efforts should be made to explore more sources for isolation of new *A. niger* strains and to awaken their silent BGCs to manufacture novel functional biomolecules using new strategies.

As one of the diseases with high mortality, cancer is one of the focuses of new drug research and development. Chen et al. discovered the fusarisetins E and F produced by a Mangrove endophytic fungus *Fusarium* sp. 2ST2 from the healthy leaves of *Kandelia candel* which showed significant cytotoxicity against human A549 cell lines with IC_50_ values of 8.7 and 4.3 μM, respectively. At the same time, other new compounds including one new chromone fusarimone A, two new benzofurans fusarifurans A and B, three new isocoumarins fusarimarins A–C were isolated, which expanded the microbial secondary metabolite pool. Lu et al. also isolated novel secondary metabolite foeniculin K with cytotoxic activity from endophytic fungus *Diaporthe foeniculina.* Unfortunately, the other ten isolated analogues foeniculins I-J did not show cytotoxic activity. Their structures were established on the basis of ^1^H and ^13^C NMR spectra together with COSY, HSQC, HMBC, and NOESY experiments. Modern pharmacological research has revealed that *Dendrobium huoshanense* has anti-inflammatory, cytotoxic, hypoglycemic and antioxidant activity. Zhu et al. studied on an endophytic *Streptomyce*s sp. HS-3-L-1 isolated from the leaves of *Dendrobium huoshanense*, and isolated three unique polyketide dimers with the cytotoxicity against MV4-11 human leukemia cell. So far only two similar natural products, strepolyketides B and C (Jiang et al., 2020) were recently reported from a marine-derived *Streptomyces*. Kang et al. discovered one new *c*-butyrolactone derivative, diaportone A, one cyclopentenone derivative, diaportone B, and one monoterpene derivative, diaportone C, along with six known compounds from endophytic fungus *Diaporthe foeniculina* BZM-15. Two of these compounds displayed significant antiproliferative effects on three human cancer cell lines (SF-268, MCF-7, and HepG2).

With the widespread use of antibiotics in clinic, bacteria gradually developed drug resistance, and this is a serious threat to the health and safety of the world. Therefore, it is of great significance to discover novel antibiotics. An original article by Ding et al. focused on the polycyclic tetramate macrolactams (PTM), and revealed that biosynthetic gene clusters (BGCs) are widespread in both Gram-positive and Gram-negative bacteria. In this study, they investigated a sponge endosymbiont *Actinoalloteichus hymeniacidonis* harboring a potential PTM-BGC. Xanthobaccin A as well as two previously reported tetramates, equisetin and ikarugamycin, exhibited antibacterial activities against *Bacillus subtilis*. In addition, these three tetramates were confirmed as metallophores for the first time. They found that all three tetramates could reduce ferric into ferrous iron, which triggers the Fenton chemistry reaction, and their antimicrobial mechanism is possibly mediated through Fenton chemistry.

Endophytic bacteria is a valuable resource pool of microorganisms with wide distribution, diverse species and diverse biological functions, and is an important source of novel compounds. In this topic, Wang et al. reported that three new humulane-type sesquiterpenoids, penirolide A, penirolide B, and 10-acetyl-phomanoxide, together with three known compounds aurasperone A, pughiinin A, and cyclo (L-Leu-L-Phe) from the endophytic fungus *Penicillium* sp. derived from the leaves of *Carica papaya* L. And four compounds penirolide B, 10-acetyl-phomanoxide, pughiinin A, and cyclo (L-Leu-L-Phe) can significantly inhibit glucagon-induced hepatic glucose production, with EC_50_ values of 33.3, 36.1, 18.8, and 32.1 μM, respectively. In response to glucagon, cAMP is a second messenger to initiate glucagon signaling cascades in hepatic glucose production. The treatment of these compounds suppressed cAMP accumulation indicated that they inhibited hepatic glucose production by suppression glucagon-induced cAMP accumulation. Liu et al. reported six new phthalan derivatives cytorhizophins D-I as well as three known derivatives cytorhizophin C, pestacin and rhizophol B from endophytic fungus *Cytospora rhizophorae*. Among them, cytorhizophins D-E and F-G were two pairs of diastereoisomers, all of them featuring a 1-phenyl-1,3-dihydroisobenzofuran scaffold with a highly oxygenated O-linked isopentenyl unit, and cytorhizophins H-I represent the first examples of phthalide family with fascinating 6/6/6/5 tetracyclic ring system fusing as unprecedented furo [4,3,2-kl]xanthen-2 (10bH)-one skeleton. Cytorhizophins D-E and F-G showed significant DPPH radical scavenging activities with EC_50_ values ranging from 5.86 to 26.80 μM, which are much better than that of the positive control ascorbic acid, they may be the promising lead compounds for the development of more effective antioxidants. Jia et al. focused on the mangrove-derived endophytes which are rich in bioactive secondary metabolites with a variety of biological activities. They isolated a fungus *Pseudofusicoccum* sp. J003 from mangrove species *Sonneratia apetala* Buch.-Ham. And then they identified a new sesquiterpenoid named acorenone C, two alkaloids, four phenolic compounds, and four steroid derivatives from this endophytic strain. Among them, acorenone C showed mild AChE inhibitory activity, with an inhibition rate of 23.34% at the concentration of 50 μM.

Wang et al. work reported that two new alkaloids tryptoquivaline Y and pseurotin I, together with eight known compounds, were isolated from *Aspergillus felis* FM324, and the fungus were purified from Hawaiian beach soil sample. One of the compounds showed weak antibacterial activity against *Staphylococcus aureus*, *methicillin resistant Staphylococcus aureus* and *Bacillus subtilis.* Two compounds inhibited NF-κB with IC_50_ values of 26.7 and 30.9 μM, respectively.

Wu et al. research selected the appropriate mutant *Aspergillus terreus* ASM-1 through the chemical mutagenesis of *A. terreus* ML-44 and resulted in the isolation of three new prenylated aspulvinones V–X, together with analogs, aspulvinone H, J-CR, and R. All the compounds were evaluated for α-glucosidase inhibitory effects with acarbose as positive control. The results showed that aspulvinones V and aspulvinone H exhibited potent α-glucosidase inhibitory activities with IC_50_ values of 2.2 and 4.6 µM in mixed-type manners and aspulvinone H significantly suppressed the increases in postprandial blood glucose levels in the C57BL/6J mice. The results suggested that aspulvinones could be promising candidates for further pharmacologic research and the mechanism of the mutagenesis of the strain ASM-1 from strain ML-44 deserve further investigation which may make contribution to understanding the metabolic regulation of aspulvinones biosynthesis.

Tuberculosis (TB) is still a global disease threatening people’s lives. Ilamycins are novel cyclopeptides with potent anti-TB activities, Li et al. focused on the preparation of ilamycin F, a major secondary metabolite isolated from the marine-derived mutant strain *Streptomyces atratus* SCSIO ZH16 *Δ*ilaR which were used as a scaffold to semi-synthesize eighteen new ilamycin derivatives (ilamycin NJL1–NJL18). Their study revealed that four of ilamycin NJLs have slightly stronger anti-TB activities against Mtb H37Rv (minimum inhibitory concentration, 1.6–1.7 μM) compared with that of ilamycin F on day 14th, but obviously display more potent activities than ilamycin F on day third, which means that these derivatives have fast-onset effect. In addition, most ilamycin NJLs had low cytotoxicity except ilamycin NJL1. These findings will promote the further exploration of structure-activity relationships for ilamycins and the development of anti-TB drugs.

This special issue also covers some biosynthesis research. Deng et al. discovered three sulfur-containing granaticin congeners, mycothiogranaticins A, B and granaticin MA from a granaticin-producing strain of *Streptomyces vietnamensis* GIMV4.0001. Gene disruptions suggested that the biosynthesis of mycothiogranaticins is mycothioldependent, providing experimental evidence for the biological origin of sulfur in this category of sulfurcontaining polyketides. And mycothiol was found to be involved in positive regulation of the biosynthesis of granaticins by maintaining the cellular redox balance. This is the first report that mycothiol can not only be a building block of polyketides but also play a regulatory role in the polyketide biosynthesis. Based on previous research that P450 Astb can dually oxidize two methyl groups (C-19 and C-21) of preasperterpenoid A to asperterpenoid A with 3-carboxyl and 11-hydroxymethyl groups, Huang et al. confirmed the oxidation order of C-19 and C-21 catalyzed by AstB, by using the combination of the quantum chemistry calculations and the experiments of obtaining the potential intermediates and the HPLC-MS detection of the potential intermediates. In the end, they revealed the catalyzed order of AstB in asperterpenoid A biosynthesis and the relationship between the oxidation stations of C-19 and C-21 in asperterpenoids and their mPTPB inhibition.

In summary, the above works presented in this special research topic illustrate the diversity of microbial natural products and highlight the importance of developing new methods to impulse the discovery of new compounds. Microorganisms are still the treasure house of new drug development in the future.
